# Early life, life course and gender influences on levels of C-reactive protein among migrant Bangladeshis in the UK

**DOI:** 10.1093/emph/eoab041

**Published:** 2021-11-27

**Authors:** Khurshida Begum, Gillian D Cooper, Nasima Akhter, Papreen Nahar, Adetayo Kasim, Gillian R Bentley

**Affiliations:** 1 Department of Anthropology, Durham University, Dawson Building, South Road, Durham DH1 3LE, UK; 2 Department of Global Health and Infection, University of Sussex, BSMS Teaching Building, Brighton BN1 9PX, East Sussex, UK; 3 Durham Research Methods Centre, Faculty of Social Sciences & Health, Durham University, Arthur Holmes Building, Durham DH1 3LE, UK; 4 UCB Pharmaceuticals, 216 Bath Road, Slough SL1 3WE, UK

**Keywords:** C-reactive protein, inflammation, early life development, gender, Bangladeshi migrants, obesity

## Abstract

**Background and objectives:**

Humans co-evolved with pathogens, especially helminths, that educate the immune system during development and lower inflammatory responses. The absence of such stimuli in industrialized countries is associated with higher baseline levels of C-reactive protein (CRP) among adults who appear at greater risk for inflammatory disorders. This cross-sectional study examined effects of early life development on salivary CRP levels in 452 British-Bangladeshis who spent varying periods growing up in Bangladesh or UK. We also analyzed how gender and central obesity modulate effects on CRP. We hypothesized that: (i) first-generation Bangladeshis with higher childhood exposure to pathogens would have chronically lower CRP levels than second-generation British-Bangladeshis; (ii) effects would be greater with early childhoods in Bangladesh; (iii) effects by gender would differ; and (iv) increasing obesity would mitigate early life effects.

**Methodology:**

Saliva samples were assayed for CRP using ELISAs, and anthropometric data collected. Participants completed questionnaires about demographic, socioeconomic, lifestyle and health histories. Data were analyzed using multiple linear regression.

**Results:**

First-generation migrants who spent early childhoods in mostly rural, unhygienic areas, and moved to UK after age 8, had lower salivary CRP compared to the second-generation. Effects differed by gender, while waist circumference predicted higher CRP levels. CRP increased with years in UK, alongside growing obesity.

**Conclusions and implications:**

Our study supports the hypothesis that pathogen exposure in early life lowers inflammatory responses in adults. However, protective effects differed by gender and can be eroded by growing obesity across the life course which elevates risks for other inflammatory disorders.

**Lay Summary:** Migrants to the UK who spent early childhoods in less hygienic environments in Bangladesh that help to educate their immune systems had lower levels of the inflammatory marker, C-reactive protein (CRP) compared to migrants who grew up in UK. Both gender and increasing obesity were associated with increased levels of CRP.

## BACKGROUND AND OBJECTIVES

C-reactive protein (CRP) is released from hepatocytes as part of the inflammatory and immune response and is regularly used as a non-specific marker of systemic inflammation [1–3]. Elevation of CRP levels can take two forms: a rapid, acute rise usually associated with a current infection or injury, or a persistent, low-grade elevation linked to conditions including obesity [4], especially visceral adiposity [5, 6], metabolic disorders, including cardiovascular diseases (CVDs) [4, 7] and type 2 diabetes mellitus (T2DM) [8, 9]. CRP is thus a useful biomarker for acute and chronic inflammatory responses [10–12], using either high sensitivity assays for plasma levels, where venepuncture is possible [1, 13] or less sensitive assays for measurement of salivary levels. The latter are easier and less invasive to collect in non-clinical settings [14], and correlate well with plasma levels [15].

Accumulating evidence of low baseline levels of CRP from small-scale populations practicing traditional lifeways suggests that the chronically elevated levels typically seen in contemporary, industrialized societies may constitute an evolutionary mismatch. The underlying theory for this mismatch relates to the ‘Old Friends’ or ‘Hygiene Hypothesis’ which argues that a reduction of immunological stimuli, particularly from soil-transmitted helminths (STH), has increased vulnerability to autoimmune diseases such as asthma, allergies and Crohn’s Disease [16–19]. Specifically, it is argued that the human immune system is highly plastic and early life experiences form and educate the adult immunological phenotype [20–23], including establishing effective T-cell and anti-inflammatory regulatory networks [17, 22, 23]. Increasing evidence also supports epigenetic mechanisms underpinning different inflammatory phenotypes [24, 25].

Bangladesh, as a semi-tropical, low- to middle-income country (LMIC) in South Asia, still has a high prevalence of infectious and parasitic diseases [26]. Many people lack access to clean water and sanitary facilities, while the quality of the health care system requires improvement [27, 28]. Helminth infections have remained high across the country in both rural and urban locations and across socioeconomic groups despite national, anti-helminthic policies since 2008 [29–31]. Drawing upon accumulating evidence for lower baseline levels of CRP among populations in similar environments [1, 32, 33], one might therefore predict that Bangladeshi adults, particularly those who grew up in rural areas and were exposed to STH during childhood, would have chronically lower levels of CRP.

Bangladesh is primarily a Muslim country with strongly gendered role expectations, particularly among the more affluent classes, which might also affect exposure to STH. In addition, many Bangladeshis, regardless of wealth, are now experiencing the dual burden of morbidity where a growing proportion are developing non-communicable diseases (NCDs) including obesity, T2DM and CVDs characterized by chronic inflammation [34, 35]. Such characteristics also hold for Bangladeshi migrants to Europe and the North American continent [36–38].

Bangladeshis in the UK, who primarily originate from the more affluent middle classes who own land in their home country and have household helpers, have had a long history of migration with third-generation children now growing up. This offers the opportunity to investigate aspects of health across generations who spent their childhoods in markedly different conditions. First-generation migrants would have experienced various portions of their lives in contrasting environments permitting examination of the effects of the childhood environment and differential exposures to pathogens on the adult phenotype, as well as effects of changing environments on characteristics associated with inflammatory disorders.

In this paper, we therefore tested the hypothesis that the environment in early life would influence levels of salivary CRP in adulthood using data from a cross-sectional study of migrant Bangladeshis in the UK. We predicted that those who were born and grew up in Bangladesh, with exposure to greater pathogen loads including STH, would have chronically lower levels of salivary CRP as adults compared to Bangladeshi migrants who spent their developmental years growing up in the UK. Second, following our earlier findings of differential effects on reproductive hormone levels in migrant adults, depending on whether they moved to the UK before or after the age of 8 [39–42], we tested the hypothesis that adult levels of salivary CRP would differ among child migrants depending on their age at migration. Specifically, we predicted that levels of CRP would be lower among child migrants who moved to the UK after 8 years of age, matching those of adult migrants, while we also predicted that levels of CRP among child migrants who moved before or at age 8 would more closely match those of the second-generation. Third, given recent discussions of how studies of CRP have paid insufficient attention to gender differences in reporting CRP levels, we tested the hypothesis that CRP levels and the effects of early life environments would differ by gender. We predicted that first-generation women would have higher levels of CRP owing to lower exposures to STH. Finally, we tested the hypothesis that lifestyle changes in the UK predisposing individuals to obesity would influence levels of CRP, predicting that increased markers of overweight/obesity would elevate levels of CRP in adulthood, and again that women would be more vulnerable to these effects.

## METHODOLOGY

### Participants

The study used cross-sectional data from a collaborative project designed to investigate relationships between migration and health among 562 first- and second-generation, Bangladeshi migrants living either in northeast England (*n* = 275) or in London (*n* = 287), and aged 25–40 years when age-related changes to CRP would be expected to be minimal [43, 44]. A subset of individuals (*n* = 479, 85%) provided saliva samples for hormonal analyses, from which a further seven participants were excluded due to inadequate or poor-quality samples, leaving a pool of 468 participants. In addition, 16 individuals with levels of salivary CRP >3000 pg/ml, above the limits of assay detection,^1^ and likely indicative of an acute infection, were removed from the dataset, leaving a final sample of 452 for this study.

Data were collected between May 2013 and April 2015. Subjects were categorized into four groups: (i) first-generation, adult migrants, born and brought up in Bangladesh, who migrated to the UK as adults aged >18 (*n* = 174); (ii) first-generation, child migrants born in Bangladesh who came to the UK aged between 9 and 18 (*n* = 73; referred to henceforth as Child Migrants >8); (iii) first-generation, child migrants born in Bangladesh who migrated to the UK aged ≤8 years (*n* = 74; referred to henceforth as Child Migrants ≤8); and (4) second-generation British-Bangladeshis who were born and brought up exclusively in the UK, with parents of Bangladeshi origin (*n* = 129). For many analyses, Groups 2 and 3 (all Child Migrants who migrated to the UK ≤18 years, *n* = 149) were combined into one, making only three groups altogether.

Exclusion criteria for the study were thyroid conditions, type I diabetes, psychosis, bipolar disorder or major depression. We also excluded women who were currently either pregnant or lactating due to the potential effect of these conditions on salivary hormone levels. In both London and northeast England, we used local Bangladeshi residents as recruiters while, in the northeast, we also employed local Bangladeshi residents as research assistants who helped with data collection including interviews. The latter were trained prior to beginning work and monitored throughout the project to ensure consistency and quality of data collection.

All participants answered a structured questionnaire that requested information about demographic and migration histories, socioeconomic, educational and environmental backgrounds, stress, health histories both recent and past, use of any current medications, dietary intake and physical exercise. Health histories included a question about any illness episodes in the last two weeks. Questionnaires were translated into Bengali and independently back-translated into English to check for accuracy. Participants were given the option of answering questionnaires in Bengali or English, but only 12 subjects in London and two in the northeast chose the Bengali questionnaire. The questionnaires were piloted extensively in draft form and modified following feedback from participants.

Anthropometric data were collected using standardized techniques [45]. Height was measured to the tenth of a cm (0.1 cm) using a portable stadiometer, weight to the tenth of a kg (0.1 kg) (according to manufacturer’s guidelines) using a portable weighing scale, and waist circumference (WC) to the tenth of a cm (0.1 cm) using a measuring tape. The body mass index (BMI) was calculated from these data. Levels of overweight and obesity conform to World Health Organisation (WHO) standards [46], while central obesity was determined using the UK National Institute for Clinical Excellence (NICE) criteria for South Asians (>0.94 cm for males and >0.80 for women) [47].

Participants followed detailed written and verbal instructions to collect and return saliva samples (collected thrice daily) and were asked to store samples in the fridge once collected. Subjects were requested to refrain from tooth brushing, betel nut chewing, eating and drinking for one hour before saliva sampling since betel nut use can be common among older first-generation adults and has been shown to interfere with other saliva sampling regimes [48]. Samples were posted to the laboratory in pre-paid envelopes the day after collection using next day delivery. On receipt at the laboratory, they were immediately frozen at −20°C until the day of assay. Collection and arrival dates were both logged.

### CRP analysis

Evening saliva samples were used to analyze CRP as the most recent relative to time spent in transit. All samples were assayed at Durham University’s Endocrinology and Ecology laboratory using commercially available salivary CRP enzyme immunoassay (ELISA) kits supplied by Salimetrics^®^ and following manufacturer’s recommended guidelines (see footnote 1). Samples were randomized across well plates to ensure all participant groups (generation, gender and region) were represented equally. If a sample was inadequate for assay or produced a value outside the calibrator range it was excluded.

On the day of assay, samples were defrosted and centrifuged (3500 rpm × 20 min at 4°C) to facilitate the separation of mucins and oral debris from the clear saliva fraction, which was aliquoted ready for assay. Saliva samples were diluted (×10) prior to assay as instructed by the manufacturer. Each saliva sample was assayed in duplicate at a volume of 50 µl per well and according to the supplied protocol. The sensitivity of the CRP assay was 10 pg/ml with a calibrator range of 93.75–3000 pg/ml. The intra- and inter-assay CVs were 8.8% and 9.1%, respectively.

### Statistical analysis

CRP data were log-transformed prior to analyses. Tertiles of CRP were also created using data for geometric mean levels. Both the categorical variable, ‘Generation’ and the numerical variable, ‘Number of Years in Bangladesh’, were used as proxy measures of exposure to environmental differences in early life. Variables were analyzed descriptively, while bivariate Pearson correlations examined initial associations between variables, Chi-square tests were used to look for differences between generations for categorical variables and *t*-tests or ANOVA for continuous variables.

Univariate analyses explored relationships between logCRP and potential demographic covariates including Generation, Age, Marital Status (married or partnered, single or separated, divorced, widowed), Gender, Number of Years Lived in Bangladesh or the UK and whether individuals grew up in a rural or urban environment. We also explored relationships with anthropometric measures (BMI, WC), measures of health (scores from the General Health Questionnaire-12) [49], physical activity (walking at least 20 min daily), socioeconomic status and an acculturation score derived from a modified Suinn–Lew Asian Acculturation Scale [50]. Variables were included in multivariate regression models if they reached a significance of 0.1. Some variables were highly significantly correlated (Age and Generation; BMI and WC; Generation and Growing Up in a Rural or Urban Location; and Number of Years Lived in Bangladesh with Number of Years Lived in the UK, both of which were also highly correlated with Age, *P* < 0.001).

The final list of covariates included in four different multiple linear regression (MLR) models were therefore Generation, Number of Years Lived in Bangladesh, and Number of Years Lived in the UK; Gender and WC appeared as additional covariates in each of the models. An initial MLR (Model 1) explored the relationship between logCRP and the categorical variable Generation (here, as three groups: Adult Migrants, All Child Migrants and the Second-Generation), and included Gender and WC as additional covariates. A second (Model 2) duplicated the first, but used the covariate Generation comprised of four groups, where Child Migrants were split into those who migrated >8 or ≤8 years. We also ran additional models using centered interaction terms to check for the potential role of mediators and moderators. A third model used the predictors, Number of Years Spent in Bangladesh, Gender and WC (Model 3a), and included an interaction term between Number of Years Spent in Bangladesh and WC, again with centered predictor variables (Model 3b). A final model used Number of Years Lived in the UK to be able to compare the potential effect of this variable separately, plus Gender and WC (Model 4). All statistics were performed using SPSS version 27, and *P*-values <0.05 were considered significant.

### Ethics

Ethical permission for the Project was granted by the Ethics Committee of the Department of Anthropology, Durham University. All participants gave informed consent, were provided with information about the Project in either English or Bengali, as appropriate, and were compensated for their time and effort. All subjects were anonymized for data entry, and data were stored in accordance with GDPR requirements.

## RESULTS

Mean age of participants was 32.9 ± 4.8 years and differed significantly by generation (ANOVA; *F*_2,__4__4__9_=28.863, *P* < 0.001); second-generation participants were significantly younger due to the history of Bangladeshi migration (Table 1). The majority of adult migrants (70%) had grown up in a rural location compared to 23% for child migrants overall, and only 2% for the second-generation (Table 1). Slightly more than half the subjects (54%) were either overweight (39%) or obese (15%). Individuals characterized by general obesity differed by generation with greater proportions among the Child Migrants ≤8 (22%) and the Second-Generation (19%) compared to Adult Migrants (13%) and Child Migrants >8 (8%; Table 1), but these differences were not significant (ANOVA, *F*_3,448_ =0.339, *P* > 0.05). While both men (41%) and women (40%) were equally overweight, significantly more women (20%) were obese compared to their male counterparts (10%; *t* = −2.509, *P* = 0.012).

**Table 1.** Descriptive statistics by generation

Variable^a^	Generation^b^					Total (*n* = 452)
	ADU (*n* = 175)	CHI > 8 (*n* = 73)		CHI < 8 (*n* = 74)	2ND (*n* = 129)	
Age^†^ (years)	33.7 ± 4.6	34.3 ± 4.6		34.1 ± 4.6	30.4 ± 4.4	32.9 ± 4.8
Gender^*^						
Male	88 (50.3)	37 (50.7)		23 (31.1)	52 (40.0)	200 (44.2)
Female	87 (49.7)	36 (49.3)		51 (68.9)	78 (60.0)	252 (55.8)
BMI	25.7 ± 3.5	25.8 ± 4.8		26.3 ± 4.9	26.4 ± 5.0	26.0 ± 4.4
Obesity^c^						
Underweight	4 (2.3)	2 (2.7)		3 (4.1)	3 (2.3)	12 (2.7)
Normal	72 (41.4)	31 (42.5)		33 (45.2)	56 (43.4)	192 (42.8)
Overweight	76 (43.7)	34 (46.6)		21 (28.8)	46(35.7)	177 (39.4)
Obese	22 (12.6)	6 (8.2)		16 (21.9)	24 (18.6)	68 (15.1)
Waist circumference	89.3 ± 8.5	87.0 ± 9.9		88.6 ± 11.4	89.5 ± 11.8	88.9 ± 10.3
Central obesity^d^						
Normal	82 (46.9)	40 (54.8)		31 (41.9)	51(39.2)	204 (45.1)
Obese	93 (53.1)	33 (45.2)		43 (58.1)	79 (60.8)	248 (54.9)
Grew up^†^						
Rural	123 (70.3)	25 (35.7)		6 (8.6)	2 (1.5)	156 (35.1)
Urban	52 (29.7)	45 (64.3)		64 (91.4)	128 (98.5)	289 (64.9)
CRP^e^ (pg/ml),** mean ± SD	293 ± 303	315 ± 475		300 ± 314	441 ± 574	340 ± 430

* *P* < 0.05; ^**^P < 0.01; ^†^*P* < 0.001.

a Presented as mean ± SD for numerical data and numbers (%) for categorical data.

b ADU = adult migrant; CHI > 8 = child migrants who moved aged over 8; CHI ≤ 8 = child migrants who moved under or equal to age 8; 2ND = Second-Generation.

c Defined according to WHO [46] criteria as ‘underweight’ <18.5; ‘normal’ = 18.5–24.9; ‘overweight’ = 25.0–29.9; ‘obese’ = >30.0.

d Defined as >80 cm for women and >94 for men [47].

e Median CRP for ADU =182.6 pg/ml, CHI >8 = 163.0 pg/ml, CHI <8 = 205.8 pg/ml, 2ND= 225.5 pg/ml, Total = 192.5 pg/ml.

A high proportion (55%) of the population also had a WC above the National Institute for Clinical Excellence (NICE, UK) cut-off for central obesity. Prevalence of central obesity differed across generations (Adult Migrants: 53%; Child Migrants >8: 45%; Child Migrants ≤8: 58%; Second-Generation: 61%; Table 1), but these differences were not significant (ANOVA, *F*_3,451_ = 1.060, *P* > 0.05). Significantly more women (75%) than men (29%) were characterized by central obesity (*t* = 2.903, *P* = 0.004). Both BMI (*r* = 0.172, *P* = 0.02) and WC (*r* = 0.287, *P* < 0.001) were significantly positively correlated with age among the Adult Migrants, but were not significantly associated with either of the child migrant groups or the Second-Generation (*P* > 0.05). Both BMI and WC increased with time spent in the UK following migration among the First Generation, as did levels of logCRP (Supplementary Figs S1 and S2), but BMI was significantly correlated with Years Lived in the UK only among the Adult Migrants (*r* = 175, *P* = 0.021). Profiles for Child Migrants >8 looked more similar to the Adult Migrants, while Child Migrants ≤8 years matched more closely to the Second-Generation (Supplementary Figs S1 and S2).

**Figure 1.**
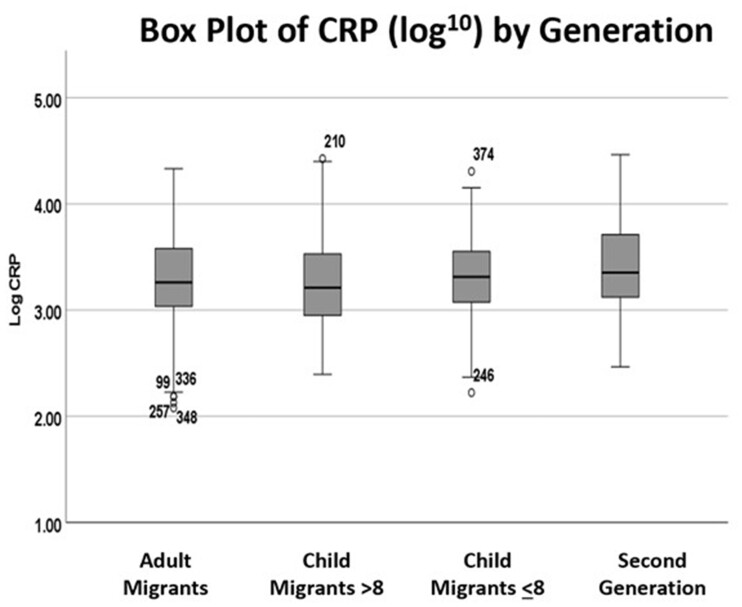
Box plot of CRP (log10) by generation

Mean salivary CRP levels for all participants was 340 (SD ± 430) pg/ml, but levels differed significantly by generation (*F*_3,4__5__1_=3.380, *P=* 0.018, Table 1, Fig. 1). When comparing CRP tertiles by central obesity (yes/no) across generations, there were significant differences among those with a normal WC (χ^2^ = 6, *n* = 204, *P* > 0.007), but none between generations for those who fell into the category of being centrally obese (*n* = 248; Supplementary Table S1). When comparing tertiles of CRP by BMI categories across generations, those with a normal BMI were close to significance (χ^2^ = 6, *n* = 204, *P* = 0.06), but the results were not significant among those who were overweight/obese (*n* = 245; Supplementary Table S1).

There was no significant correlation between Age and logCRP levels for either the Adult Migrants (*r* = 0.131, *P* = 0.09), Child Migrants >8 (*r* = −0.86, *P* = 0.47) or Child Migrants ≤8 (*r* = 0.47, *P* = 0.69), but the correlation was significant and negative for the Second-Generation (*r* = −0.209, *P* = 0.02). LogCRP was significantly positively correlated with Number of Years Lived in the UK (*r* = 0.095, *P* < 0.05), and significantly negatively correlated with Number of Years Lived in Bangladesh (*r* = −0.125, *P* = 0.008). There were no significant associations between logCRP and individuals walking more than 20 min each day, self-assessed financial situation or net household income.

Unadjusted Model 1, evaluating whether Generation predicted logCRP levels, and where Child Migrants formed one group, was highly significant (*r*^2^ = 0.021; *F*_2,451_ = 4.801, *P* = 0.009). It showed that Adult (*t* = −2.728, *P* = 0.007) and Child (*t* = −2.736, *P* = 0.006) Migrants had significantly lower logCRP levels compared to the Second-Generation (Table 2). After adjusting for Gender and WC, the Model was still highly significant (*r*^2^ = 0.092; *F*_4,451_ = 11.323, *P* = <0.001) and significant differences remained between Adult Migrants and the Second-Generation (*t* = −2.400, *p* = 0.017) as well as Child Migrants and the Second-Generation (*t* = −2.532, *P* = 0.012; Table 2). The adjusted model also revealed that females (*t* = 4.524, *P* < 0.001) had significantly higher logCRP compared to males, while logCRP increased significantly with a higher WC (*t* = 4.373, *P* < 0.001; Table 2).

**Table 2.** Multiple regression models predicting CRP by covariates

	Unadjusted Estimate		Adjusted Estimate	
Model 1	B	95% CI	B	95% CI
Variables	Unadjusted^**^		Adjusted^†^	
Generation				
Adult migrants	−0.131^**^	−0.226, −0.037	−0.112^*^	−0.204, −0.020
Child migrants	−0.136^**^	−0.234, −0.038	−0.122^*^	−0.217, 0.027
Second-generation	Ref		Ref	
Female			0.174^†^	0.098, 0.249
Male	Ref			
Waist circumference			0.008^†^	0.004, 0.012

Model 2				
Variables	Unadjusted^*^		Adjusted^†^	
Generation				
Adult migrants	−0.123^**^	−0.217, −0.029	−0.103^*^	−0.195, −0.012
Child migrants > 8	−0.157^**^	−0.276, −0.038	−0.118^*^	−0.234, −0.002
Child migrants < 8	−0.089	−0.207, 0.030	−0.096	−0.211, 0.018
Second-generation	Ref		Ref	
Female			0.172^†^	0.095, 0.248
Male	Ref			
Waist circumference			0.008^†^	0.004, 0.012

Model 3a				
Variables	Unadjusted^**^		Adjusted^†^	
Number of years lived in Bangladesh	−0.005^**^	−0.009, −0.001	−0.004^*^	−0.008, 0.000
Female			0.165^†^	0.088, 0.241
Male	Ref			
Waist circumference			0.008^†^	0.005, 0.012

Model 3b				
Variables	Unadjusted^†^		Adjusted^†^	
Number of years lived in Bangladesh	−0.005^*^	−0.009, −0.001	−0.005^*^	−0.008,−0.001
Female	Ref		0.162^†^	0.086, 0.237
Male				
Waist circumference			0.010^†^	0.006, 0.014
*Centred interaction term*				
WC by number of years lived in Bangladesh			0.001^**^	0.000, 0.001

Model 4				
Variables	Unadjusted^*^		Adjusted^†^	
Number of Years Lived in the UK	0.004^*^	0.000, 0.008	0.002	−0.001, 0.006
Female			0.171^†^	0.094, 0.248
Male	Ref			
Waist circumference			0.008^†^	0.005, 0.012

* *P* < 0.05;

** *P* < 0.01;

† *P* < 0.00.

**Figure 2.**
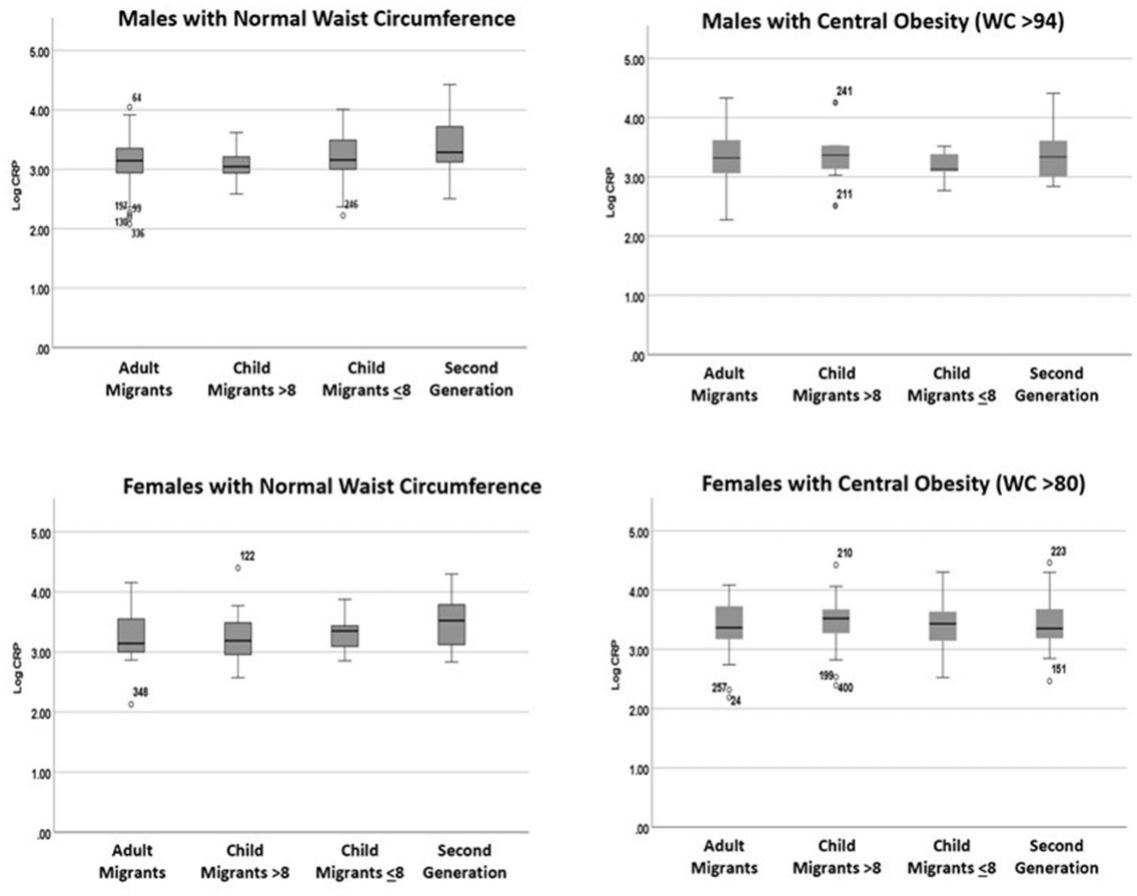
Boxplots of CRP (log10) by generation, gender and waist circumference

For the unadjusted results in Model 2, Generation (using four groups) again significantly predicted variation in logCRP levels (*r*^2^ = 0.02; *F*_3,451_ = 3.041, *P* = 0.03). Both Adult Migrants (*t* = −2.562, *P* = 0.01) and Child Migrants >8 (*t* = −2.585, *P* = 0.01) were associated with significantly lower logCRP levels compared to the Second-Generation, while Child Migrants ≤8 were not (*t* = −1.470, *P* = 0.142; Table 2). After adjusting for Gender and WC, the model remained highly significant (*r*^2^ = 0.089, *F*_3,451_ = 8.742, *P* < 0.000). Again, both Adult Migrants (*t* = −2.220, *P* = 0.03) and Child Migrants >8 (*t* = −2.005, *P* = 0.05) were significant predictors in the model relative to the Second-Generation, but not Child Migrants ≤8 (*t* = −1.649, *P* >0.05), while both Gender (*t* = −4.424, *P* < 0.001) and WC (*t* = 4.365, *P* <.001) were highly significant predictors of logCRP levels (Table 2). Figure 2 reveals that individuals with central obesity looked similar in their logCRP levels regardless of Generation, while it was those with a normal WC who reflected generational, early life differences in logCRP levels.

We further explored the potential role of both WC and Gender as either mediators or moderators of the effect of early years spent in Bangladesh proxied by Generation. In relation to the former, WC was not significantly correlated with Generation (*r* = 0.01, *P* = 0.831), suggesting it is not a mediator. Furthermore, a model where a centered interaction term between WC and Generation was added to the MLR using just these two variables as predictors for logCRP levels was significant overall (*r*^2^ = 0.064, *F*_7,451_ = 4.344, *P* < 0.000). However, the interaction removed any significance for WC in the main model (*t* = 1.098, *P* = 0.273), while retaining significance for Adult Migrants (*t* = −2.711, *P* = 0.007) and Child >8 (*t* = −2.505, *P* = 0.013). The only significant interaction term was WC * Adult Migrants (*t* = 2.414, *P* = 0.016), suggesting WC is a weak moderator at best, despite this model being significant overall (Supplementary Table S2, Model S1).

In relation to Gender, this variable was significantly correlated with Generation (*r* = 0.111, *P* = 0.018). Gender also acted as a highly significant predictor of logCRP in a model with just Generation (four groups) as an additional covariate (*r*^2^ = 0.050, *F*_4,447_ = 5.925, *P* < 0.000) (Supplementary Table S2, Model S2). These results suggest Gender acts as a partial mediator of the effects of Generation on logCRP. When an interaction term between Gender and Generation was added to this regression model, the model remained highly significant (*r*^2^ = 0.064, *F*_7,444_ = 4.368, *P* < 0.000), but Gender was no longer significant in the main part of the model (*t* = −0.114, *P* = 0.909). The interaction terms for Gender * Adult Migrants (*t* = 2.170, *P* = 0.031) and Gender * Child >8 (*t* = 2.122, *P* = 0.034) were significant, while Gender * Child ≤ 8 was not (*t* = 1.709, *P* = 0.088; Supplementary Table S2, Model S2).

Given these results, we then ran Models 1 and 2 again separately by Gender including WC as a covariate along with Generation (Supplementary Table S3, Models S3a, S3b, S4a and S4b). Adjusted Model S3a, selecting for males only (*n* = 200) and using three groups for Generation, was significant overall (*r*^2^ = 0.104, *F*_3,196_ = 7.609, *P* < 0.001) with Adult Migrants (*t* = −2.879, *P* = 0.004), Child Migrants (*t* = −3.049, *P* = 0.003) and WC (*t* = 2.975, *P* = 0.003) all as significant covariates. Results for just males were similar for Model S4a (using four groups for Generation) with the adjusted Model being significant overall (*r*^2^ = 0.105, *F*_4,195_ = 5.691, *P* < 0.001), and Adult Migrants (*t* = −2.873, *P* = 0.005), Child Migrants > 8 (*t* = −2.775, *P* = 0.006), Child Migrants ≤ 8 (*t* = −2.177, *P* = 0.031) and WC (*t* = 2.943, *P* = 0.004) as significant covariates. However, when we selected for just women (*n* = 252) in the dataset, both Models S3b (*r*^2^ = 0.039, *F*_3,248_ = 3.328, *P* < 0.02) and Model S4b (*r*^2^ = 0.037, *F*_4,247_ = 2.381, *P* < 0.052) were significant overall, WC remained a significant covariate (Model 4a: *t* = −3.051, *P* = 0.003; Model 4b: *t* = −3.057, *P* = 0.002), but neither Adult Migrants or Child Migrants were significant predictors.

Unadjusted results for Model 3a (Table 2), using Years Lived in Bangladesh as the dependent variable, showed that this Model was highly significant (*r*^2^ = 0.016, *F*_1,450_ = 7.096, *P* < 0.001) and revealed a significant relationship between this variable and logCRP (*t* = −2.664; *P* = 0.008). The adjusted Model was still highly significant (*r*^2^ = 0.086, *F*_3,448_ = 14.053, *P* < 0.001), and Years Lived in Bangladesh remained significant (*t* = −2.197; *P* = 0.029), while Gender (*t* = 4.292; *P* < 0.000), and WC (*t* = 4.572; *P* < 0.000) were likewise significant covariates. In Model 3b, which added a centered interaction term between Years Lived in Bangladesh and WC, the model was still highly significant (*r*^2^ = 0.103, *F*_4,447_ = 12.892, *P* < 0.001) with significant covariates of Years Lived in Bangladesh (*t* = −2.521, *P* = 0.01), Gender (*t* = 4.193, *P* < 0.000) and WC (*t* = 5.202, *P* < 0.000). The interaction term was also significant (*t* = 2.956, *P* = 0.003).

Finally, Model 4 used Years Lived in the UK as a predictor variable (Table 2). The unadjusted model was significant (*r*^2^ = 0.009, *F*_1,450_ = 4.137, *P* = 0.043) and showed a significant relationship between logCRP and the independent variable (*t* = 2.034, *P* = 0.043), with more Years in the UK associated with higher levels of logCRP. When the Model was adjusted for Gender and WC, the Model was highly significant (*r*^2^ = 0.079, *F*_3,448_ = 12.858, *P* < 0.001), while Years Lived in the UK was no longer significant (*t* = 1.251, *P* > 0.05), but both Gender (*t* = 4.384, *P* < 0.000) and WC (*t* = 4.439, *P* < 0.000) were highly significantly associated with logCRP.

## CONCLUSIONS AND IMPLICATIONS

In recent years, the association between degrees of exposure to infections in early life and persistent low-grade inflammation has been increasingly studied, particularly in relation to risks for chronic diseases in later life [1, 2, 32]. The argument has been that education of the immune system during early life and frequent exposure to pathogens serves to regulate activation and deactivation of the immune system and inflammatory responses [1, 20–23, 51, 52]. Individuals who lack such exposures may be unable to downregulate inflammation effectively, potentially explaining chronically elevated levels of CRP among many individuals who develop in environments with lower microbial exposures typical of highly industrialized societies [2, 53].

McDade et al. [1] for example, measured levels of CRP using a high specificity plasma assay among participants in the Cebu Longitudinal Health and Nutrition Survey in the Philippines where infectious and parasitic diseases are still high compared to northern hemisphere countries. They found that elevated levels of microbial exposures in childhood were associated with chronically lower levels of CRP as an adult. Similarly, low chronic levels of CRP were found among the indigenous Shuar of Ecuador who practise a mixed subsistence strategy of horticulture, fishing and hunting, and who are exposed to high levels of diseases in their tropical, Amazonian environment [2, 33]. Both of these studies focused on populations that remained in their natal environments as adults.

A more recent study, using dried blood spots, examined CRP levels from a small sample (*n* = 31) of Ecuadorian migrants in the USA who originated from the same region as the Shuar and who had migrated mostly as adults (*n* = 28), defined in this study as aged >13 [32]. While examining the hypothesis that higher exposure to pathogens in early life would lower baseline levels of CRP among these migrants, the authors also took serial measures of CRP to measure inter-individual variability across a period of two months. As predicted, median levels of CRP in these migrants were low relative to other populations, while both BMI and gender predicted CRP levels in multiple variable regression models.

Here, in our paper, we used a larger, multi-generational sample of 452 British-Bangladeshis, first testing the hypothesis and associated prediction that early life exposure to greater immunological challenges would lower adult levels of salivary CRP and that there might be a particular period in early life which had greater effect. We also explored the role of gender in influencing early life exposures and logCRP predicting that women would have higher levels. Since migrant Bangladeshis in the UK and other industrialized countries are at increasing risk of inflammatory disorders such as overweight/obesity, T2D and CVDs [54–56], we predicted that more years spent in the UK would increase levels of CRP. Our hypotheses and predictions were supported: those individuals who had grown up in Bangladesh had lower levels of logCRP with greater effects observed if they had spent their early childhood (before age 8) in that country. Furthermore, gender appeared to mediate these effects in women who, as a group, did not show a significant impact of the early life environment on levels of logCRP and who were also more vulnerable to obesity across the life course. In general, spending more years in the UK with accumulating levels of obesity increased levels of inflammation.

Although we do not have direct measures of STH exposure among the study participants, the majority of first-generation migrants (77%) had grown up in rural areas of Bangladesh where evidence suggests they would have been exposed to a wide range of infectious and parasitic diseases compared to second-generation British-Bangladeshis who grew up in the UK [28, 57]. Participants in the study were aged 25–40 at recruitment, meaning most would have been children during the late 1970s and into the 2000s, depending on their age at recruitment. During those decades, 96% of the rural Bangladeshi population had access to tubewells for drinking purposes, but hygiene and sanitation in the country were far less developed relative to today [58]. For example, in 1985, only 4% of the rural population had access to a sanitary latrine, rising to 26% in 1991, while 35% had unhygienic latrines (e.g. uncovered pits, ‘bucket’ or open latrines over water), and 39% of people used open defecation [58]. The prevalence of STH in Bangladeshi children also remained high up until 2005 when a survey estimated that 80% of those examined were infected with at least one kind of STH [31]. In a more recent study [59], half the people tested in Bangladesh were still positive for STH, while the rate was up to 90% among those living in the worst conditions.

Furthermore, children were still not widely vaccinated against measles, tetanus, polio, diphtheria, whooping cough and tuberculosis until the Bangladeshi government launched the WHO Expanded Programme of Immunization in 1979, raising country-wide levels of vaccination from 2% in 1984, to 81% in 2000 and >95% in 2019 [60, 61]. Associated with these statistics were estimates that 35% of child mortality in Bangladesh was attributable to diarrheal diseases in 1991, reflecting unhygienic environments and exposure to pathogenic microbes [58]. Moreover, a recent study of drinking water quality in Bangladesh, using data from 2012 to 2013, still showed medium levels of contamination in households owning chickens and/or cattle that would likely have exposed individuals to diarrheal diseases [62]. Matters would have been worse in the last millennium.

In our regression models, both Generation (Models 1 and 2) and Number of Years Lived in Bangladesh (Model 3) were significant predictors of logCRP levels, with lower CRP among individuals who had spent their childhoods in Bangladesh, when presumably exposures to pathogens might have had a critical impact on the developing immune system, particularly in early childhood. Specifically, child migrants who had spent their first eight years in Bangladesh aligned with the adult migrants when predicting lower logCRP levels, while child migrants who moved to the UK before age 8, were more similar to the second-generation British-Bangladeshis. In contrast, the number of years spent in the UK did not significantly predict logCRP levels when accounting for gender and WC.

These results lend support to accumulating evidence from studies in evolutionary medicine that humans co-evolved with STH and that such exposures, especially during early life, modulate inflammatory responses [1, 2, 32, 33]. Two unanswered questions, however, have been: (i) how gender might be a significant factor in differentiating among these early life and life course exposures; and (ii) the extent to which life course exposures to other inflammatory conditions, including overweight and obesity, could override the potentially protective effects of a better-educated immune response acquired during development.

In relation to the first point concerning gender, Klein and Flanagan [63] have recently discussed the absence of reporting results by sex in studies of immunological function (amounting to <10% in the literature), sex referring to biologically based differences between females and males. Similarly, Clough [64] noted that proponents of the Hygiene Hypothesis have not paid sufficient attention to gender variation that might result in different vulnerabilities to STH and resulting health across the life course. This also appears to be replicated in some of the more anthropological studies relating to CRP levels among different populations [1, 32, 33]. Here, gender refers to differences in both culture and behavior that reflect specific expectations placed on girls/boys and women/men.

Both Gender and WC were highly significant covariates in Models 1–3 and increased the ability to predict logCRP by 7% in adjusted Models 1 and 2. Mediation analyses of both variables and their effects on Generation as predictors of logCRP levels in Model 2 showed that Gender is a partial mediator of logCRP level and that WC may be a weak moderator. Higher CRP levels are known to be related to greater central adiposity [65], and this is a particular risk factor for women [5, 66], as well as South Asians [4]. The women who participated in the research here were much more likely to be obese than men, and a higher proportion were also characterized by central obesity [38]. It may be that Bangladeshi women are much more susceptible to an increase in overweight and obesity following migration to the UK, with consequences for biomarkers of inflammation. This might then override any potential protective effect of early life education of the immune system. In addition to central adiposity, generalized overweight and obesity are accompanied by chronic inflammation reflected in higher CRP levels [67–69]. The mechanisms whereby obesity leads to increased CRP levels are not clearly understood, but inflammatory cytokines such as IL-6 are produced by adipose tissue [68], and this process is exacerbated in conditions of increased central adiposity.

The relationship between gender and CRP levels is controversial across epidemiological studies. Data from European populations have shown no significant differences between females and males [70–72], but two large multi-ethnic studies reported that women had higher CRP levels irrespective of ethnic background [73, 74]. Studies of south-east Asian groups reported that Korean [75] and Japanese [74] men had higher CRP levels compared to women, while no gender differences were reported by Ye et al. for Chinese subjects [76]. Although the reasons for inconsistencies in gender difference in CRP concentrations are not clearly understood, differences in lifestyles and metabolic risk factors between men and women may be responsible. A meta-analysis of CRP demonstrated interactions between obesity and elevated CRP levels that were more marked among women than men, and also among populations from Europe and the United States [69].

In the study here, Gender was consistently a significant predictor of logCRP. When Models 1 and 2 were run separately by Gender, Generation was no longer a significant predictor for women but this was not the case for men, while WC remained significant in models for both. Klein and Flanagan’s [64] observations, discussed above, of a dearth of reporting results by sex in the immunological literature is, in fact, mirrored in the relevant evolutionary literature. For example, the variable ‘gender’ was a significant predictor of CRP levels in the study of Ecuadorian migrants by Shattuck-Heidorn et al. [32], but McDade et al. [1] did not include this variable as a predictor in their model for the Cebu population in the Philippines. Our findings highlight the need to shed more light on sex differences in susceptibilities to inflammatory disorders among future such studies [64].

Alternatively, or in addition, there may be gendered differences in the ways in which middle class, Bangladeshi girls and boys are exposed to STH in early life, with potential for boys to have greater exposure if they are allowed more freedom to play outside or get dirty [64]. Existing data for infection with STH among Bangladeshi children in their home country are almost wholly confined to the poor whose lives are different from the middle classes. In just one paper, however, where children aged 0–15 years from different socioeconomic backgrounds and living conditions were compared [77], there were significant differences in prevalence of STH infection among the different groups ranging from 81% in children who lived in the worst housing to only 18% among those who lived in the highest quality housing. These differences were mirrored when children were grouped by family income. Huq and Shaikh [77] also noted that infections were higher among boys than girls. These data, and others for Bangladesh [78, 79], suggest that infections among children up to about 4–5 years old are higher for boys with the situation reversing between genders at about adolescence. These findings might imply that the immune system of boys is either more exposed to STH during early childhood, or reflects gender differences in the response to such exposures. Boys are also known to have much higher rates of respiratory infections than girls during childhood and these might also influence the ways in which the immunological system develops during early life [80].

In relation to how life course exposures might modify the potential educational effect on the immune system, Shattuck-Heidorn et al. [32] proposed from their findings of Ecuadorian migrants in the USA that the generally low levels of CRP in this population point to a *life-long* benefit of developmental regulation of immune function, but their sample was extremely small and included only one migrant group. In an earlier paper, McDade et al. [81] had compared their results for high-sensitivity CRP levels from the Cebu Longitudinal Study (when participants were aged 20–22) with data from 616 young adults aged 19–24 from the US National Health and Nutrition Examination Survey. The goal was to assess the impact of markers of adiposity, including WC, on levels of CRP in these populations. Overall, the Cebu sample had low plasma levels of baseline CRP, as described earlier in their 2010 study [1] and lower anthropometric indices compared to the US sample. In both the Cebu and US groups, both gender and WC predicted CRP, similar to findings in our study here. However, the Cebu sample had significantly lower plasma CRP levels for matched WC measurements compared to the US sample, even where these were comparatively high (>70 cm). In the Bangladeshi groups presented here, CRP levels were similar across the generations among individuals above the cut-off for central obesity, and it is only those individuals with a normal WC where significant differences emerged by developmental environment (Fig. 2).

One potential issue with McDade et al.’s [81] results was that the US samples were assayed in a different laboratory possibly introducing inter-laboratory error. The participants were also sufficiently young that it would be difficult to predict longer-term effects of obesity on CRP levels. Our study here, however, compared older individuals aged 25–40 from the same source population in Sylhet, northeast Bangladesh, some of whom had changed environments and had spent variable amounts of time in the UK. This age range is still young enough that increases in CRP would likely reflect effects of inflammatory conditions such as overweight/obesity as opposed to independent age effects [43, 44]. Indeed, the only group to show a positive correlation between age and CRP was the second-generation while, conversely, their BMI and WC were high but not correlated with age.

All samples were collected using the same protocols and were assayed in the same laboratory. While first-generation migrants had lower levels of salivary CRP compared to the second-generation overall, the data showed that overweight, and particularly high central obesity, appeared to mitigate the beneficial impact of the early life environment on CRP levels. Despite the effects of rising indicators of obesity, levels of CRP among Adult Migrants and Child Migrants >8, on average, were never as high as those among the second-generation British-Bangladeshis and Child Migrants ≤8. These differences across the generations could provide valuable information to health practitioners when assessing health risks among Bangladeshis and South Asian migrants considered at greater risk for inflammatory disorders compared to some other ethnic groups.

It remains to be seen how rising levels of overweight and obesity within LMICs like Bangladesh will also affect levels of CRP across the life course, particularly among those who continue living in areas with high pathogen exposures. At present, Bangladesh like the Philippines and many other countries, suffers from the dual burden of morbidity with a variety of chronic diseases resulting from rapid development [82–84]. This development has been accompanied by lifestyle changes promoting obesogenic environments for which existing health systems are ill-prepared [85], and which can exist alongside persistent poverty [86–90].

Chronically elevated levels of CRP are an independent risk factor for several morbidities including CVDs [91, 92], T2DM [8], the metabolic syndrome [93], stress [94] overall frailty [95], and mortality among the elderly [4, 56], but are also symptomatic of several of these conditions. Relative to other South Asian countries like India and Pakistan, rates for T2DM in Bangladesh are higher; approximately 8% of the urban population and 2-3% of the rural population in Bangladesh were estimated to suffer from T2DM in 2006 [96], while absolute numbers of people with T2DM are expected to more than triple in Bangladesh by 2030 [97]. These morbidities are expected to impact significantly on health budgets [52]. Understanding the role of the childhood environment in influencing later life health, particularly in specific, early parts of childhood, alongside the role of gender in influencing environmental exposures, and a greater appreciation of evolutionary influences on our phenotypes, may therefore help in the development of strategies to mitigate these increasing health problems.

### Study strengths and limitations

The study is cross-sectional and compared salivary CRP levels across Bangladeshi groups in north and south UK. All of the migrant population originated from Sylhet region in northeast Bangladesh, came from the same socioeconomic stratum and were uniformly Muslim, allowing for control of various cultural factors that might otherwise affect CRP levels. Participants were also drawn from limited geographic areas in London and northeast England thereby controlling for place of residence in the UK. Saliva samples were taken using the same sampling framework for all individuals and assayed in the same laboratory using standardized techniques. Collection of a large amount of qualitative and quantitative data allowed us to control for potential covariates while exploring potential explanatory variables.

The study relied on one sample for measurements of CRP rather than repeated samples which would have permitted intra-individual and more long-term comparisons, as advocated by McDade and others [98, 99]. However, at least in environments with low exposure to infectious diseases, levels of CRP are relatively stable within individuals [100]. Less is known about the validity of high specificity CRP levels in saliva compared to plasma, although such measures have been increasingly used in recent years as well as representing a less invasive method of data collection [101]. A majority of studies show good correlation (*r* = 0.72) with high specificity plasma measures of CRP [15].

Given the labour-intensive methods of data collection for this study, participants were asked to mail their samples back to the laboratory. Some samples may therefore have been in transit slightly longer than others which may have affected sample quality. We attempted to control for this by only assaying the evening sample which would have been in transit for the least amount of time. Ouellet-Morin et al. [15] found a 12–14% decline in salivary CRP values across a 24–96 h delay in freezing when samples were kept at room temperature, but potential waiting and transit times affected all our samples equally and there is unlikely to be any form of systematic bias between groups in this respect. It does mean, however, that levels of CRP reported here are likely to be lower overall than if we had been able to freeze samples immediately after collection.

## SUPPLEMENTARY DATA

Supplementary data is available at *EMPH* online.

## DATA ACCESSIBILITY

The data are available at the Durham Research Online Data Repository: doi:10.15128/r1gx41mh89z.

## Supplementary Material

eoab041_Supplementary_Data

## Footnotes

1 Salimetrics, the company that supplied the CRP ELISA kit have since introduced a new assay but instructions and specifications for the assay used at the time of analyses can be found at: https://fnkprddata.blob.core.windows.net/domestic/data/datasheet/SAL/1-3302.pdf
