# Supplementary material for: Early life, life course and gender influences on levels of C-reactive protein among migrant Bangladeshis in the UK
Source: Evol Med Public Health. 2021 Nov 27;10(1):21–35. doi: 10.1093/emph/eoab041 (PMC8754477; doi:10.1093/emph/eoab041)
Supplement: eoab041_Supplementary_Data [file eoab041_supplementary_data.zip › SupplementaryFigureLegends.docx]

**Supplementary Figure 1**

*Proportions of Adult Migrants and Child Migrants >8 in different Categories of BMI, WC and CRP Tertiles by Length of Time in the UK (median time =10 years for ADU and 24 years for CHI>8)*

**Supplementary Figure 2**

*Proportions of Child Migrants Who Migrated <8 Years and Second-Generation British-Bangladeshis in different Categories of BMI, WC and CRP Tertiles by Length of Time in the UK (Median Years in UK =30 for Child Migrants and by Age in Second-Generation)*
