# Supplementary material for: Early life, life course and gender influences on levels of C-reactive protein among migrant Bangladeshis in the UK
Source: Evol Med Public Health. 2021 Nov 27;10(1):21–35. doi: 10.1093/emph/eoab041 (PMC8754477; doi:10.1093/emph/eoab041)
Supplement: eoab041_Supplementary_Data [file eoab041_supplementary_data.zip › SupplementaryTables.docx]

**Supplementary Table S1**. Descriptive variables by CRP tertiles

| **Variables^1^** | **Lower tertile^2^** | **Middle tertile^3^** | **Upper tertile^4^** | **Total** |
| --- | --- | --- | --- | --- |
| *Generation* |  |  |  |  |
| Adult Migrants | 61 (40.7) | 59 (39.1) | 54 (35.8) | 174 (38.6) |
| Child Migrants > 8 | 31 (42.5) | 19 (26.0) | 23 (31.5) | 73 (16.3) |
| Child Migrants < 8 | 23 (31.1) | 26 (35.1) | 25 (33.8) | 74 (16.5) |
| Second-Generation | 34 (22.7) | 46 (30.5) | 49 (32.5) | 129 (28.6) |
| Age | 33.0 ± 5.1 | 32.6 ± 4.6 | 33.1 ± 4.7 | 32.9 (4.8) |
| *Gender*† |  |  |  |  |
| Male | 88 (58.7) | 58 (38.4) | 54 (35.8) | 200 (44.2) |
| Female | 62 (41.3) | 93 (61.6) | 97 (64.2) | 252 (55.8) |
| BMI† | 25.0 ± 4.0 | 26.1 ± 3.8 | 26.9 ± 5.1 | 26 (4.4) |
| *Obesity*† |  |  |  |  |
| Underweight | 6 (4.0) | 3 (2.0) | 3 (2.0) | 12 (2.7) |
| Normal | 77 (51.7) | 58 (38.7) | 57 (38.0) | 192 (42.8) |
| Overweight | 52 (34.9) | 71 (47.3) | 54 (36.0) | 177 (39.4) |
| Obese | 14 (9.4) | 18 (12.0) | 36 (24.0) | 68 (15.1) |
| Waist Circumference* | 86.6 ± 9.2 | 89.1 ± 10.1 | 90.9 ± 11.1 | 88.9 (10.3) |
| *Central obesity* |  |  |  |  |
| Normal*** | 85 (56.7) | 63 (41.7) | 56 (37.1) | 204 (45.1) |
| Obese | 65 (43.3) | 88 (58.3) | 95 (62.9) | 248 (54.9) |
| *Grew up* |  |  |  |  |
| Urban | 89 (61.0) | 96 (64.4) | 104 (69.3) | 156 (35.1) |
| Rural | 57 (39.0) | 53 (35.6) | 46 (30.7 | 289 (64.9) |

**p*<.01; †*p*<.001;

^1^ Presented as mean ± SD for numerical data and numbers (%) for categorical data

^2^Low tertile = CRP <135.7 pg/mL; ^3^Medium tertile = CRP 135.7 - 294.9 pg/mL; ^4^High tertile =CRP>294.9 pg/mL

***Supplementary Table 2.*** *Multiple regression models to check for moderation by WC and Gender with logCRP as the dependent variable.*

| *MODEL S1* | **Adjusted Estimate**  **Without Interaction Term**  B 95% CI | **Adjusted Estimate**  **With Interaction Term**  B 95% CI |
| --- | --- | --- |
| **Variables** |  |  |
| Centered Waist Circumference | 0.007† 0.003, 0.011 | 0.003 -0.003, 0.009 |
| *Generation*  Adult Migrants  Child Migrants >8  Child Migrants <8  Second-Generation | -0.122** -0.215, -0.029  -0.140* -0.258, -0.022  -0.083 0.166, -0.200  Ref | -0.128** -0.221, -0.035  -0.151* -0.269, -0.032  -0.085 -0.201, 0.032  Ref |
|  |  |  |
| Interaction Adult Migrants*Centered WC |  | 0.011* 0.002, 0.021 |
| Interaction Child >8* Centered WC |  | -0.001 -0.012, 0.010 |
| Interaction Child <8* Centered WC |  | 0.004 -0.006, 0.014 |
|  |  |  |
| *MODEL S2* |  |  |
| **Variables** |  |  |
| Gender | 0.148† 0.071, 0.225 | -0.008 -0.151, 0.135 |
| *Generation*  Adult Migrants  Child Migrants >8  Child Migrants <8  Second-Generation | -0.107* -0.200, -0.014  -0.140* -0.258, -0.023  -0.101 -0.219 0.016 | -0.226** -0.366, -0.086  -0.283† -0.455, -0.111  -0.236* -0.436, -0.035 |
|  |  |  |
| Interaction Adult Migrants*Gender |  | 0.207* 0.020, 0.395 |
| Interaction Child >8* Gender |  | 0.255* 0.019, 0.490 |
| Interaction Child <8* Gender |  | 0.215 -0.032, 0.461 |

**p*<.05; ***p*<.01; †*p*<.001

***Supplementary Table 3.*** *Multiple regression models predicting CRP by Generation run separately for males and females*

| *MODEL S3a (Males)* | **Unadjusted Estimate**  B 95% CI | **Adjusted Estimate**  B 95% CI |
| --- | --- | --- |
| **Variables** | Unadjusted** | Adjusted† |
| *Generation*  Adult Migrants  Child Migrants  Second-generation | -0.226** -0.369, -0.083  -0.265** -0.420, -0.110  Ref | -0.206** -0.346, -0.065  -0.237** -0.390, -0.084  Ref |
| Waist circumference |  | 0.009** 0.003, 0.015 |
|  |  |  |
| *MODEL S3b (Females)* |  |  |
| **Variables** | Unadjusted | Adjusted* |
| *Generation*  Adult Migrants  Child Migrants  Second-Generation | -0.033 -0.157, 0.091  -0.050 -0.172, 0.073  Ref | -0.040 -0.162, 0.082  -0.044 -0.165, 0.077  Ref |
| Waist circumference |  | 0.007** 0.003, 0.012 |
|  |  |  |
| *MODEL S4a (Males)* |  |  |
| **Variables** | Unadjusted** | Adjusted† |
| *Generation*  Adult Migrants  Child Migrants >8  Child Migrants <8  Second-Generation | -0.226** -0.370, -0.083  -0.283** -0.459, -0.107  .236* -0.441, -0.031  Ref | -0.206** -0.347, -0.065  -0.246** -0.420, -0.071  -0.222* -0.424, -0.021 |
| Waist circumference |  | -.009** 0.003, 0.015 |
|  |  |  |
| *MODEL S4b (Females)* |  |  |
| **Variables** | Unadjusted | Adjusted* |
| *Generation*  Adult Migrants  Child Migrants >8  Child Migrants <8  Second-Generation | -0.019 -0.142, 0.104  -0.029 -0.187, 0.130  -0.021 -0.163, 0.121  Ref | -0.027 -0.148, 0.094  -0.017 -0.173, 0.140  -0.022 -0.161, 0.118  Ref |
| Waist circumference |  | -0.007** 0.003, 0.012 |

**p*<.05; ***p*<.01; †*p*<.001
