# Supplementary figures and images for: Early life, life course and gender influences on levels of C-reactive protein among migrant Bangladeshis in the UK
Source: Evol Med Public Health. 2021 Nov 27;10(1):21–35. doi: 10.1093/emph/eoab041 (PMC8754477; doi:10.1093/emph/eoab041)

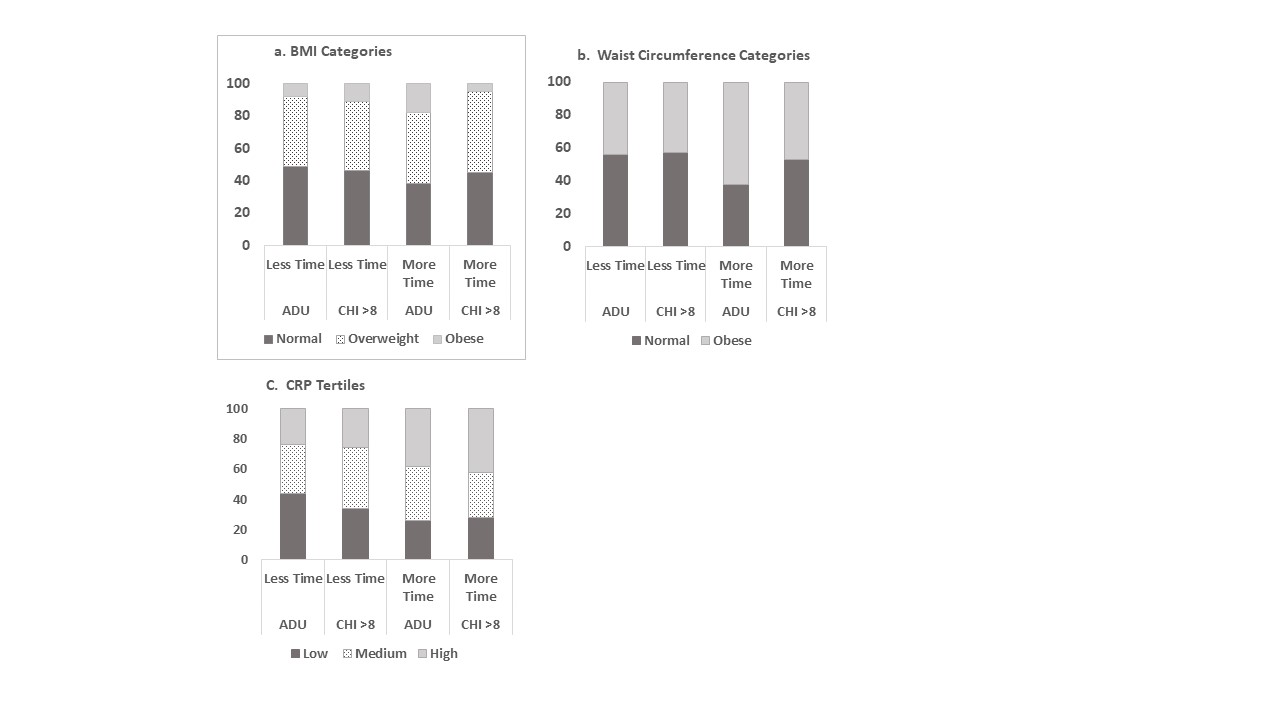

Supplement: eoab041_Supplementary_Data [file eoab041_supplementary_data.zip › SupplementaryFigure1.jpg]

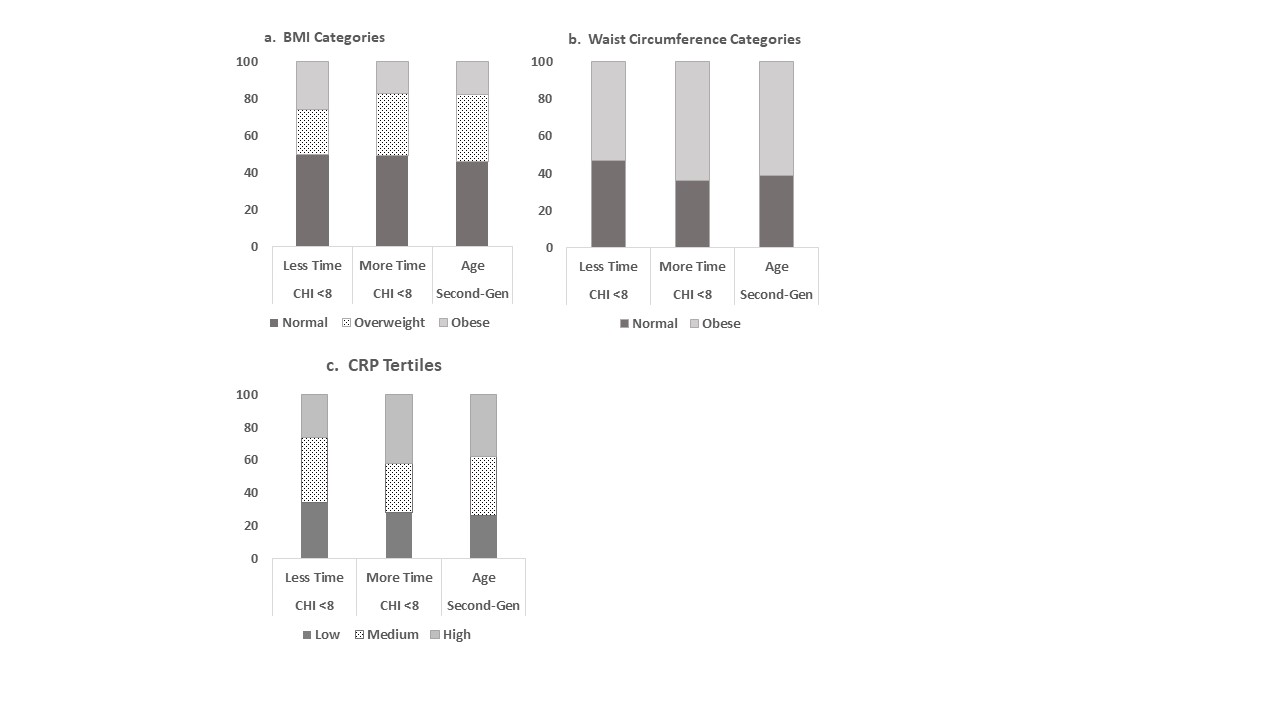

Supplement: eoab041_Supplementary_Data [file eoab041_supplementary_data.zip › SupplementaryFigure2.jpg]
